# ViT-FuseNet: Same-Patient MRI–Pathology Feature Fusion for Multimodal Breast Cancer Diagnosis

**DOI:** 10.3390/jcm15145486

**Published:** 2026-07-13

**Authors:** Birgül Karahan, Merve Parlak Baydoğan, Serpil Ağlamış, Seda Arslan Tuncer, Aslı Özer Zeren

**Affiliations:** 1 Surgical Medical Sciences, Faculty of Medicine, Firat University, Elazig 23119, Türkiye; 2 Computer Technologies, Technical Sciences Vocational School, Firat University, Elazig 23119, Türkiye; mpbaydogan@firat.edu.tr; 3 Internal Medicine, Faculty of Medicine, Firat University, Elazig 23119, Türkiye; drserpil23@yahoo.com.tr (S.A.); asliozer321@gmail.com (A.Ö.Z.); 4 Software Engineering, Faculty of Engineering, Firat University, Elazig 23119, Türkiye; satuncer@firat.edu.tr

**Keywords:** breast cancer, multimodal imaging, pathology images, MRI, vision transformer, deep learning, feature extraction, classification

## Abstract

**Background:** In breast cancer diagnosis, while radiological imaging modalities provide important insights into the structural characteristics of tumors, pathological examinations remain essential for establishing a definitive diagnosis. **Methods:** This study proposes a Vision Transformer (ViT)-based approach developed by fusing magnetic resonance imaging (MRI) and pathology images from the same patient in breast cancer diagnosis. In the study, models combined with different classifiers were trained using ViT-16 and ViT-32 architectures. Performance of the models was evaluated using accuracy, F1 score, sensitivity, precision, ROC, AUC, and Precision–Recall (PR) curves. **Results:** The findings show that models multimodal image fusion models outperform single-modality models in accuracy, precision, and sensitivity, demonstrating that the fusion approach is an effective method for breast cancer diagnosis. Specifically, the ViT-B/32 (Fusion) + SVM model proved to be the most successful, achieving 92.53% accuracy and a PR-AP value of 0.9708. **Conclusions:** These results demonstrate that evaluating radiological and pathological images improves diagnostic accuracy and reliability, and that multimodal image fusion is effective in distinguishing malignant lesions from benign ones.

## 1. Introduction

Breast cancer remains the most common cancer among women worldwide and a leading cause of cancer-related deaths. Early and accurate diagnosis of this disease is vital for developing effective treatment strategies and improving patient outcomes [1,2].

Mammography has long been considered the gold standard in breast cancer screening and diagnosis, proving effective in detecting tumors, microcalcifications, and other abnormalities. However, mammography faces limitations, such as shadowing of lesions, particularly in dense breast tissue, which can lead to diagnostic errors. To overcome these challenges, Magnetic Resonance Imaging (MRI) has been introduced into clinical practice, offering high sensitivity, especially in the detection of invasive cancers [3,4]. 

In diagnosis, radiological imaging methods provide critical information about the localization, size, and spread of lesions, while pathological examination plays a fundamental role in making a definitive diagnosis and determining the biological characteristics of the tumor.

The combined evaluation of radiology and pathology increases diagnostic accuracy and contributes to reducing false-negative or false-positive results [5,6].

Artificial intelligence (AI) and deep learning (DL) techniques have shown great potential as decision support systems in medical imaging. While traditional Convolutional Neural Networks (CNNs) are effective in extracting local features in images, ViT-based architectures, which have gained popularity recently, are capable of capturing global dependencies and long-range relationships in images thanks to their attention mechanisms [7,8]. However, most AI applications are still based on a single data modality, and single imaging approaches can be limited by factors such as tumor heterogeneity, tissue complexity, and the inability to capture different biological features simultaneously [9,10].

In clinical practice, radiologists evaluate information from multiple different sources together, not just a single type of imaging, when making decisions [11]. Data fusion methods, which combine these different information sources into a single coherent representation, significantly increase the diagnostic sensitivity of DL models. Data fusion aims to maximize the discriminatory power of the model by combining complementary information. This method is critical for reducing diagnostic errors, especially minimizing false positives (FP) and false negatives (FN) [12,13].

Two different ViT architectures were used in this study. The proposed multimodal fusion approach aims to improve classification performance by combining the structural context provided by MRI images with the cellular information provided by pathology images under a single unified representation. In this context, this study aims to contribute to the literature on multimodal medical image analysis and to offer a viable solution for clinical decision support systems.

The main contributions of this study to the literature are presented below:

- A multimodal deep learning-based diagnostic approach using MRI and pathology images from the same patient is proposed.
- A feature-level fusion-based system is developed to integrate deep features obtained from different image types.
- Through the proposed fusion approach, macroscopic structural information (MRI) and microscopic cellular information (pathology) are evaluated simultaneously, significantly improving diagnostic accuracy.
- The developed method offers a more distinctive and richer feature representation compared to single-modality-based systems. Combining pathology and MRI medical image data from the same patients has significantly improved diagnostic performance by overcoming the limitations of single approaches.
- To our knowledge, there are no studies in the literature that use MRI and pathology images from the same patients for breast cancer diagnosis. Furthermore, a novel dataset is introduced to the literature.
- The performance of the proposed system was evaluated under different ViT patch sizes, and the effectiveness of the method was demonstrated through comparisons with traditional ViT models.

### Literature Review

The literature shows that many studies have been conducted on the diagnosis and detection of breast cancer using both unimodal, multimodal, and fusion-based methods. A number of these studies are presented in this section.

Varshney et al. presented a hybrid classification approach to improve the performance of breast cancer diagnosis in small datasets. In the study, deep learning features and shape descriptors from the ResNet50 model were combined with traditional hand-made features derived from Gray-Level Co-Occurrence Matrix (GLCM) features. The SVM classifier achieved the highest performance with 96.88% accuracy [2]. Jing et al. trained the ResNet-34 deep learning model with Maximum Density Projection (MIP) data of both the left and right breasts from 837 MRIs. The developed model achieved an Area Under Curve (AUC) of 0.81, 98% sensitivity, and 98% negative predictive value (NPV) [14]. Lo Gullo et al. focused on breast lesion classification. While visual assessment based on the Breast Imaging Reporting and Data system (BI-RADS) provided only 53.4% accuracy, the developed machine learning model demonstrated a much superior diagnostic performance with 81.5% accuracy, 91.4% specificity, and 80.0% positive predictive value [15]. In the study by Melekoodappattu et al., an ensemble approach was developed for the detection of breast cancer in digital mammography images. The study combined a modified CNN and a tissue feature-based approach using the DDSM dataset. As a result, 97.9% accuracy and 98.3% specificity were obtained [16]. Singh et al. comparatively evaluated the discrimination levels of different tissue and geometric features in the classification of breast masses using the k-nearest neighbor (k-NN) classifier approach. In the study, the k-NN classifier yielded the best result, achieving 90.4% accuracy, 92.0% sensitivity, and 88.0% specificity [17]. Yaqub et al. proposed a two-stage intelligent breast cancer diagnosis system based on mammography images. In the study, the adaptive Trans-Res-UNet (ACA-ATRUNet) enhanced with Atrous Convolution and attention mechanisms was used for tumor segmentation, and the multi-scale DenseNet-based ACA-AMDN model was used for classification; approximately 89% accuracy and 88–89% sensitivity were obtained in the MIAS and CBIS-DDSM datasets, significantly reducing the misclassification rates [18]. In the study proposed by Lee et al., the aim was to perform breast cancer classification in DBT images using a transformer-based deep neural network. Experimental findings increased the AUC value from 0.88 to 0.91, the sensitivity from 81.0% to 87.7%, and the specificity from 80.5% to 86.4% in the test set [19]. A summary of the related studies is presented in Table 1.

A review of recent literature reveals that multimodal approaches are becoming increasingly widespread. However, a large portion of existing studies is based on combining histopathological and radiological images (e.g., mammography or ultrasound) obtained from different databases [21]. Although these studies offer a multimodal analysis framework, it is noteworthy that the datasets used are mostly not composed of paired images from the same patient, but rather are created by integrating independent data sources [22,23,24,25]. To our knowledge, no study that explicitly uses histopathological and MRI images obtained from the same patient together exists in the literature. This situation shows that most existing multimodal approaches are based on the combination of heterogeneous datasets and that the use of integrated multimodal data at the real patient level is still a significant research gap.

The primary motivation for the proposed study is to provide a more comprehensive diagnostic assessment by combining two different imaging modalities. Accordingly, macroscopic features such as the tumor’s anatomical structure, size, and spread to surrounding tissues are obtained through MRI images, while microscopic cellular structure, histopathological characteristics, and malignancy grade are obtained through pathology images. The integration of information from both levels provides a more in-depth and holistic analysis for tumor diagnosis and classification.

## 2. Materials and Methods

This section provides detailed information about the datasets and methods used in breast cancer diagnosis.

### 2.1. Materials

The dataset used in this study was collected and labeled by physicians within the scope of clinical imaging processes conducted at Elazığ Firat University Research Hospital. In creating the dataset, MRI images were first recorded for each participant, and then pathology images were obtained for the same patient for histopathological examination. Thus, a matched and comparable multimodal data structure belonging to two different image modalities was created. Detailed descriptive information regarding the dataset used in the study, the numerical distributions of the images, and modality characteristics are presented in Table 2.

**Figure 1:**
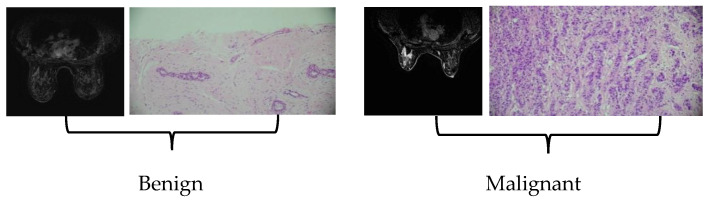
Sample images of the dataset used.

In the dataset, MRI and pathology images belonging to the same class (benign or malignant) were matched at the patient level, and an independent fusion sample was obtained from each match. As a result of this process, a total of 1207 fusion samples were created, of which 633 were benign and 574 were malignant. Therefore, the sample numbers reported in experimental analyses and performance evaluations represent the fusion samples created at the patient level, not the number of patients. In this study, MRI and pathology images were used in their raw format. Modality-specific advanced preprocessing steps (e.g., MRI slice selection or regional filtering) were not applied. All images were only resized to fit the model input and subjected to standard normalization processes. This approach ensured that data from different modalities were made suitable for the model and comparable. Examples of MRI and pathology images from the multimodal dataset are presented in Figure 1.

### 2.2. Methods

In this study, the ViT architecture was used for deep feature extraction from MRI and pathology images of the same patients for breast cancer diagnosis. In the feature extraction process, ViT-B/16 and ViT-B/32 models were applied separately to evaluate the representational power of different patch sizes. Each image was processed with two different ViT models to obtain high-level feature representations.

#### 2.2.1. Vision Transformers Models

ViT processes the input image by dividing it into fixed-size patches. The total number of patches obtained from the image is calculated as shown in Equation (1).

(1) $$\;N=\frac{H\times W}{P^{2}}$$

Here, *H* and *W* refer to the height and width of the image, respectively, and P refers to the patch size. After the resulting patches undergo linear embedding, they are transferred to the transformer encoder along with positional coding. The self-attention mechanism, which is the basic component of ViT, is presented in Equation (2).

(2) $$Attention(Q,\;K,\;V)=Softmax\left(\frac{{QK}^{T}}{\sqrt{d_{k}}}\right)\;V$$

In Equation (2), *Q*, *K*, and *V* refer to the query, key, and value matrices, respectively, and *d_k_* refers to the size of the key vector. Thanks to this mechanism, the model creates distinctive feature representations by learning the relationships between image patches.

#### 2.2.2. Proposed Hybrid Model and Evaluation

**Figure 2:**
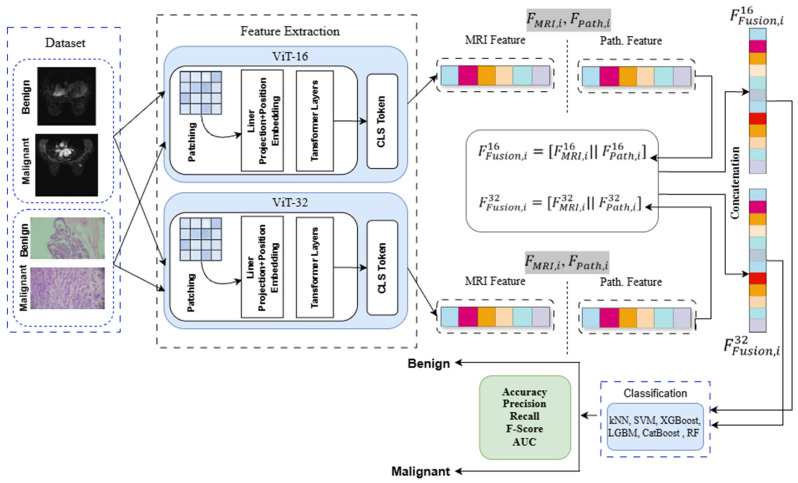
General architecture of the proposed multimodal breast cancer diagnostic system. The arrows indicate the data flow within the proposed framework, while the colored blocks schematically represent the extracted feature vectors.

The ViT-16 model produces more detailed local representations by dividing the images into 16 × 16 patches, while the ViT-32 model offers a more global context through 32 × 32 patches [26]. In this way, it was aimed to reflect structural and textural information at different scales in the model. In addition, a concatenated multimodal feature representation was created using a fusion approach with MRI and pathology feature vectors obtained from the same ViT variant. Thus, high-level feature vectors were obtained. The working architecture of the proposed system is presented in Figure 2.

Step 1: A multimodal dataset labeled with benign/malignant class labels was obtained by matching MRI and pathology images of the same patient. All MRI and pathology images were resized to 224 × 224 pixels, converted to tensor format, and normalized using ImageNet mean and standard deviation values to provide a suitable input structure for the pre-trained ViT model. Data augmentation was not applied in this study.

Step 2: Deep feature vectors based on classification token (CLS) tokens were extracted by separately feeding MRI and pathology images into ViT-B/16 and ViT-B/32 models.

Step 3: Modality-specific features were concatenated using a patient-level, feature-level fusion approach to create a unified multimodal feature representation. MRI and pathology images of the same patient were matched according to their class labels (benign/malignant). A one-to-one matching strategy was adopted between the images in both modalities for each patient. For each matched image pair, feature vectors were extracted separately using a pre-trained ViT model. The resulting features were concatenated at the feature level to create a single fusion representation. This approach prevents unnecessary sample duplication in the dataset while preserving patient-level variation, contributing to the model’s learning process.

Step 4: Benign and malignant classification was performed for four different models using ViT-B/16, ViT-B/32, ViT-B/16-Fusion, and ViT-B/32-fusion features. Each resulting feature vector was associated with its corresponding patient ID. This allowed for patient-based data splitting during the model evaluation phase.

Step 5: At this stage, the deep feature vectors obtained with the ViT model were analyzed using machine learning-based classifiers. All classification models were trained only on the training dataset, and performance evaluations were carried out on independent test datasets separated on a patient-by-patient basis. This approach prevented samples from the same patient from being used in both the training and testing processes, thus preventing data leakage.

Step 6: The performance of the developed methods was analyzed using Accuracy, Precision, Recall, F1-score, PR-AP, and ROC-AUC metrics.

As shown in Figure 2, the feature extraction process was performed independently for each patient’s image modality. First, the patient’s MRI image was processed using the ViT model, and a MRI-specific feature vector was obtained. Then, the pathology image of the same patient was processed using the same ViT model, and a pathology-specific feature vector was extracted. The hyperparameter settings for the model and classification algorithms used in this study are presented in detail in Table 3.

This process was applied separately for both ViT-16 and ViT-32 models. Thus, for each patient, feature representations belonging to two different modalities corresponding to each ViT variant were obtained. Mathematically, this process is given in Equations (3)–(6).

(3) $$F_{MRI,i}^{16}=\varphi_{ViT16}(X_{MRI,i})$$

(4) $$F_{MRI,i}^{32}=\varphi_{ViT32}(X_{MRI,i})$$

(5) $$F_{Path,i}^{16}=\varphi_{ViT16}(X_{Path,i})$$

(6) $$F_{Path,i}^{32}=\varphi_{ViT32}(X_{Path,i})$$

**Figure 3:**
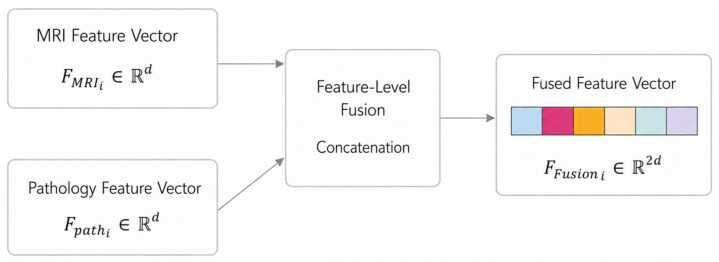
Mathematical representation of the fusion process at the feature level. The arrows indicate the data flow, and the colored blocks schematically represent the feature vectors.

Here, i denotes the patient index;$\;X_{CT,i}$ and $X_{Path,i}$, i represent the MRI and pathology images of the i-th patient, respectively; $\varphi_{ViT16}\;$ and $\varphi_{ViT32}$ denote the feature extraction functions of the ViT-16 and ViT-32 models; and $F^{16}$ and $F^{32}\;$ indicate the extracted deep feature representations. The feature-level fusion process defined by Equation (7) is illustrated schematically in Figure 3.

(7) $$F_{Fusion,i}=[F_{MRI,i}||\;F_{Path,i}]$$

Feature vectors $F_{MRI,i}$, $F_{Path,i}$ obtained from MRI and pathology images of the same patient are combined using a concatenation operation to create a combined feature representation called $F_{Fusion,i}$.

In this context, MRI and pathology feature vectors obtained from the same ViT variant were concatenated to form a single common representation. The ViT-16 based fusion process is given in Equation (8).

(8) $$F_{Fusion,i}^{16}=[F_{MRI,i}^{16}||F_{Path,i}^{16}]$$

This equation shows that the MRI and pathology images of the patient—i were processed using the ViT-16 model with a 16 × 16 patch size. As a result of this process, a 2000-dimensional composite feature vector was obtained for each patient. The same fusion process was applied to the ViT-32 model, creating ViT-32-based composite feature representations. The ViT-32-based fusion process is given in Equation (9).

(9) $$F_{Fusion,i}^{32}=[F_{MRI,i}^{32}||\;F_{Path,i}^{32}]$$

The features obtained with this equation represent more global contextual information thanks to the larger patch size of the ViT-32 model [27].

With this developed model, the macroscopic structural information provided by MRI images and the microscopic cellular information provided by pathology images for each patient are concatenated under a single fused feature representation using two different ViT models. As a result of this process, a 2000-fused feature vector is obtained in each ViT-16 and ViT-32 model. This fused feature vector simultaneously contains the complementary information of both modalities and is used in the classification phase. Fused feature representations were fed into ViT-16- and ViT-32-based classifiers to distinguish between benign and malignant breast lesions. Thanks to this structure, the contribution of complementary information obtained from both different image modalities and ViT models with different patch sizes to the classification performance is analyzed. This approach is referred to as multimodal feature-level fusion in the literature. In the fusion process, MRI and pathology images from the same patient were matched using a one-to-one matching strategy to create multimodal data pairs.

The number of matched samples created for each patient was determined based on the minimum number of images in each modality. This is shown in Equation (10):

(10) $$N_{paried}^{\left(i\right)}=min\left(N_{MRI}^{\left(i\right)},\;N_{Path}^{\left(i\right)}\right)$$

Here, $N_{MRI}^{\left(i\right)}$ and $N_{Path}^{\left(i\right)}$ represent the number of MRI and pathology images for the i-th patient, respectively. This approach ensures a balanced matching between both modalities and prevents overrepresentation of either modality. Where the number of images differed between modalities, excess images were randomly selected and excluded from the dataset, avoiding any data duplication. Matching was only performed between images belonging to the same class (benign–benign, malignant–malignant), thus preventing inconsistent matches between classes [28].

The classification processes of the proposed system were performed using kNN, Support Vector Machines (SVM), XGBoost, Light Gradient Boosting Machine (LGBM), CatBoost, and Random Forest (RF) algorithms, and the obtained results were examined comparatively. Furthermore, to prevent data leakage and enhance methodological robustness, the dataset was divided into patient-level components: 80% training and 20% testing. This ensured that all samples for each patient were placed within a single dataset (training or testing). Following this patient-level data splitting operation, each sample was assigned to the relevant subset of data based on the patient ID.

This approach ensures that each sample is assigned to the relevant cluster based solely on the patient it belongs to, guaranteeing that all samples belonging to the same patient are included in a single subset of the data. This effectively prevents patient-level data leakage by ensuring that no patient is present in both the training and test sets.

In the analysis of the classification results, a confusion matrix was used, which reveals the correct and incorrect classifications of the model in detail. The accuracy, sensitivity, precision, specificity, and F1-score performance values of the system were calculated from the confusion matrix of each model [29]. In addition to conventional evaluation metrics, Matthews Correlation Coefficient (MCC) and Cohen’s Kappa Coefficient (κ) were calculated to provide a more comprehensive statistical assessment of the proposed model. MCC evaluates the overall classification performance by considering all elements of the confusion matrix, while Cohen’s Kappa measures the agreement between predicted and true labels beyond chance [30].

## 3. Experimental Results

Through experimental analysis, this section comprehensively evaluates the performance of the multimodal feature-level fusion approach proposed for breast cancer diagnosis. The experimental procedures were conducted in two different scenarios. In the first scenario, the feature representations obtained from the ViT-16 and ViT-32 models were directly classified to examine their performance. In the second scenario, the MRI and pathology feature representations (ViT-16 and ViT-32) from the same patient were concatenated at the feature level, and the resulting concatenated representations were used in the classification phase. In both scenarios, classification processes were performed using kNN, SVM, XGBoost, LGBM, CatBoost, and RF algorithms, and the obtained results were examined comparatively. In this way, both the feature extraction capabilities of the ViT-16 and ViT-32 models and the contribution of the feature-level fusion approach to classification performance were systematically evaluated.

In this study, six experimental pipelines were evaluated to assess different data modalities and model architectures. Two Vision Transformer variants (ViT-B/16 and ViT-B/32) were used to test three configurations: MRI-only classification, pathology-only classification, and a multimodal approach combining MRI and pathology images. The results of the first scenario are presented in Table 3. The Average Precision (AP) value obtained from the PR curve summarizes the model’s ability to distinguish the positive class across different threshold values.

Table 4 shows that the highest performance among pathology image-based models was obtained with ViT-B/32 + LGBM (AP = 0.9074). However, MRI image-based models generally offered higher performance. Specifically, the ViT-B/16 + XGBoost model (AP = 0.9495) yielded the best result in the study. In terms of accuracy, the highest value was obtained with the ViT-B/16 + MRI + Random Forest model (Accuracy = 0.8838).

Table 5 shows a remarkable performance improvement achieved through the fusion of both pathology and MRI images. Experiments conducted by combining deep features obtained from MRI and pathology images showed a significant performance increase compared to single-modality models. Specifically, the ViT-B/32-based fusion model with the SVM classifier (AP = 0.9708) achieved the highest performance in the study. This result demonstrates that the two different image modalities contain complementary information and significantly improve classification performance when used together. Similarly, other powerful classifiers such as LGBM (AP = 0.9696) and XGBoost (AP = 0.9683) also offer high performance values, supporting the consistency of the fusion approach. Furthermore, a significant improvement in overall accuracy was achieved with the CatBoost classifier in the ViT-B/16-based fusion model, yielding an Accuracy of 0.9046. These findings demonstrate that combining these two modalities creates a richer and more distinctive feature space, since MRI images represent macro-level tissue structure and pathology images represent micro-level cellular details.

MCC and κ were calculated to more comprehensively evaluate the classification performance of the proposed models. Table 5 shows that the MCC and κ values are above 0.90 for all fusion-based models. This indicates that, considering all components in the complexity matrix, the proposed models have high classification success and an “almost perfect agreement” level between the predicted classes and the actual classes. In particular, the ViT-base-patch32-224 Fusion + XGBoost model (MCC = 0.9589, κ = 0.9586) exhibits the highest statistical performance, supporting the effectiveness of the fusion approach at the proposed feature level.

**Figure 4:**
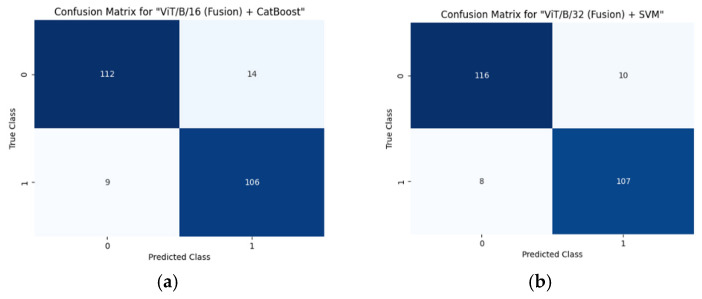
Confusion Matrices of the Highest Success Fusion Classifiers Obtained with ViT-base-patch16-224 (**a**) and ViT-base-patch32-224 (**b**) Models.

Confusion matrices were created for each model and classifier to allow for detailed interpretation of the classification results. Figure 4 shows the confusion matrices of the highest-performing fusion classifiers obtained with the ViT-base-patch16-224 and ViT-base-patch32-224 models.

**Figure 5:**
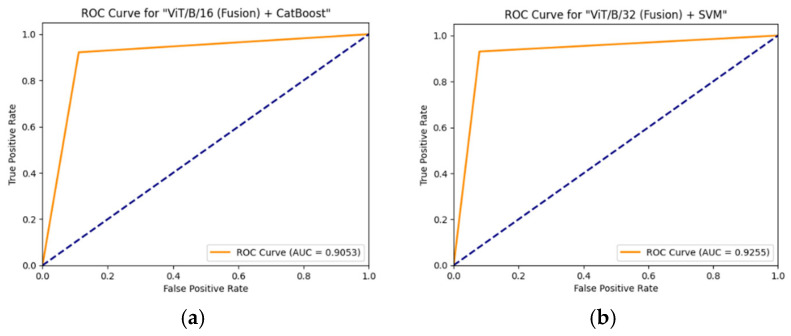
ROC Curves of ViT/B/16 (Fusion) + CatBoost (**a**) and ViT/B/32 (Fusion) + SVM Models (**b**). The dashed diagonal line represents the performance of a random classifier.

When Figure 4 was analyzed, the FP and FN values for the ViT-B/16 + CatBoost model are obtained as 14 and 9, respectively, and for the ViT-B/32 + SVM model as 10 and 8, respectively. The ROC curves for these models are presented in Figure 5.

**Figure 6:**
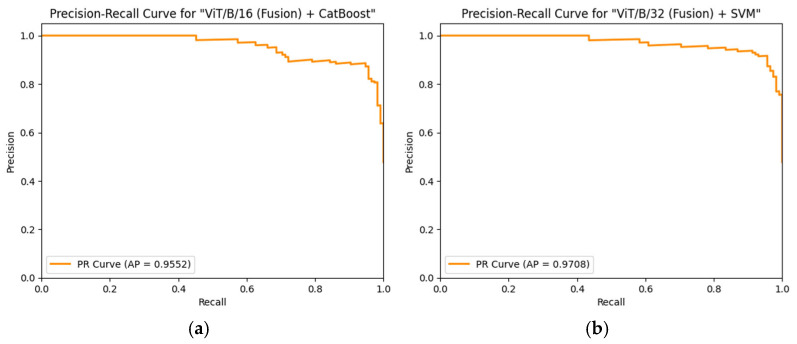
PR Curves of ViT/B/16 (Fusion) + CatBoost (**a**) and ViT/B/32 (Fusion) + SVM (**b**) Models.

When Figure 5 is analyzed, the AUC value obtained for the ViT-B/16 + CatBoost model is 0.9053, and for the ViT-B/32 + SVM model it is 0.9255. The PR curves for these models are given in Figure 6.

When Figure 6 is analyzed, the AP value is obtained as 0.9552 for the ViT-B/16 + CatBoost model and 0.9708 for the ViT-B/32 + SVM model.

## 4. Discussion

This study proposes a novel approach using Transformer-based ViT models for breast cancer diagnosis and investigates the effect of fusion of pathology and MRI images with large-scale feature vectors on model sensitivity and accuracy. Fusion of these two image types was observed to significantly improve classification performance by providing multi-level information integration in breast cancer diagnosis. Furthermore, the preference for a feature-level fusion strategy minimized information loss by preserving the distinctive features of each modality. The results demonstrate that the fusion method significantly improves model performance compared to single-modality approaches. In particular, the ViT-B/32 + SVM and ViT-B/16 + CatBoost combinations stood out with low error rates and high AUC values. ROC curves and confusion matrices revealed that the models exhibited balanced and reliable performance in terms of both sensitivity and specificity.

When the confusion matrices of the fusion (MRI + pathology)-based models are evaluated, both models are seen to perform classification with high accuracy. However, the ViT-B/32 + SVM model producing lower FP and FN values indicates that it offers a more balanced and reliable performance compared to the ViT-B/16 + CatBoost model. In particular, the low false negative rate reveals that malignant cases are detected more successfully, providing a significant clinical advantage (Figure 4).

Examining the ROC curves (Figure 5), both fusion models exhibit high discriminative performance. However, the ViT-B/32 + SVM model achieving a higher AUC value indicates that the model is more successful in separating benign and malignant classes. Furthermore, these results are consistent with the low FP and false FN rates obtained in the confusion matrices. This suggests that the ViT-B/32 architecture can learn multimodal feature representations more effectively (Figure 5).

When the PR curves are evaluated (Figure 6), it is seen that both models maintain high sensitivity values over wide recall intervals. The Average Precision (AP) value obtained from the PR curve summarizes the model’s ability to distinguish the positive class at different threshold values. In this context, the fact that the ViT-B/32 + SVM model has a higher AP value indicates that it exhibits a more successful performance in detecting malignant cases compared to the ViT-B/16 + CatBoost model. The high AP values obtained support the fact that the proposed multimodal fusion approach can provide reliable and effective results even in unbalanced datasets (Figure 6).

### Limitations and Future Works

The proposed model has some limitations:

- The datasets used were obtained from specific sources and were not validated with multi-center datasets from different centers; this may limit generalizability.
- Only MRI images and pathological features were used in the study; clinical information such as age, genetic profile, treatment history, and family history were not included in the evaluation.
- Feature-level fusion was performed with a fixed structure, and the contributions of the modalities were not dynamically weighted.
- Combining high-dimensional feature vectors increases computational cost and memory requirements.
- Only pre-trained ViT-based models were used in the study; different transformer architectures and multimodal DL approaches were not evaluated.

In this context, it is suggested that future development and expansion of the study should focus on the following aspects:

- Generalizability of models should be evaluated using multi-center and balanced datasets from different institutions.
- More comprehensive decision support systems can be developed by integrating patient history, biomarkers, and genetic information into multimodal data fusion.
- Clinical reliability of decisions can be strengthened by using methods that increase the explainability of models.
- Studies can be conducted on dynamically learning the contribution of modalities using attention mechanisms or adaptive weighting strategies.
- Finally, it is recommended that the developed system be tested in clinical use scenarios and optimized with physician feedback.

## 5. Conclusions

A multimodal and patient-centric feature-level fusion framework is introduced in this study to enhance breast cancer diagnosis by integrating complementary information from MRI and pathology images. Accordingly, deep feature maps were generated using ViT architectures with different patch sizes and classified using six different algorithms. The accuracy of the proposed system increased through the fusion approach, which effectively leveraged both the visual feature extraction power of the ViT architecture and the complementary information from multiple image sources. The findings demonstrate that the strong visual feature extraction capacity of ViT-based models and the integration of different image types can yield significantly more successful results compared to single-source models. The proposed method reveals an effective approach for developing a highly accurate and reliable decision support system for critical health problems such as breast cancer. Furthermore, the fact that a solution focused on an MRI + Pathology fusion approach for breast cancer diagnosis has not yet been presented in the literature adds significant originality to this study in terms of designing an alternative solution. This research presents a novel approach for breast cancer diagnosis in the fields of medical image processing and deep learning, and emphasizes the importance of AI-assisted systems in healthcare.

## Figures and Tables

**Table 1:** Summary of Related Studies.

Ref. No	Authors	Data Type	Method	Performance Metrics
[2]	Varshney et al.	Breast Images	ResNet50 + GLCM + Shape Features + SVM	Accuracy: 96.88%, Recall: 97.12%, Precision: 96.54%, F-Score: 96.83%
[14]	Jing et al.	Breast MRI	ResNet-34	AUC: 0.81, Sensitivity: 98%, NPV: 98%
[15]	Lo Gullo et al.	Breast MRI	Radiomics + Machine Learning	Accuracy: 81.5%, Specificity: 91.4%, PPV: 80.0%, NPV: 82.1%
[16]	Melekoodappattu et al.	Digital Mammography	Modified CNN + Texture Features	Accuracy: 98%, Specificity: 97.8%
[17]	Singh et al.	Mammography	k-Nearest Neighbor (k-NN)	Accuracy: 90.4%, Sensitivity: 92.0%, Specificity: 88.0%
[18]	Yaqub et al.	Mammography (MIAS, CBIS-DDSM)	ACA-ATRUNet + ACA-AMDN	Accuracy: ≈89%, Sensitivity: ≈88–89%
[19]	Lee et al.	Digital Breast Tomosynthesis (DBT)	Vision Transformer	AUC: 0.88, Sensitivity: 81%, Specificity: 80.5%
[20]	Abdullah et al.	Breast MRI	Different deep Learning Methods	AUC: 0.90, Sensitivity: 88%, Specificity: 90%

**Table 2:** Image Modalities and Quantitative Distributions in the Dataset.

Feature	MRI Images	Pathology Images
Patient Group Distribution	35 Benign/ 34 Malign	35 Benign/ 34 Malign
Number of Patients	69	69
Distribution of classes	633 Benign/ 574 Malign	633 Benign/ 574 Malign
Total Number of Images	1207	1207
Modality Type	2D cross-sectional images	Microscopic tissue images

**Table 3:** Hyperparameter specifications of the implemented models.

Models/Algorithms	Parameters/Metrics/Characteristics
ViT-B/16 and ViT-B/32	Input Size: 224 × 224 × 3, Patch Size: 16 × 16 and 32 × 32, Transformer Layers: 12, Attention Heads: 12, MLP Dimension: 3072, Pretrained on ImageNet, other parameters: default
Feature Vector Dimension	1000 for per modality, fused representation: 2000 (concatenation)
Activation Function (MLP)	GELU/Used to introduce non-linearity
Attention Normalization	Softmax/Used to normalize attention weights
kNN, SVM, XGBoost, LGBM, CatBoost, RF	k value: 5, distance metric: minkowski, distance weight: equal, kernel: linear, kernel scale: automatic, gamma: scale, degree: 3, C parameter: 1, Estimators:100, criterion: gini, var_smoothing: 1 × 10^−9^, min_samples_split:2, min_samples_leaf:1, classification type: binary, other parameters: default

**Table 4:** Experimental results on the classification of MRI and pathology images based on ViT-16 and ViT-32 models.

ViT Model	Classifier	Performance Evaluation Criteria							
		Accuracy	Precision	Recall	F-Score	AUC	AP	MCC	(κ)
Vit-base-patch16-224 Only Pathology	KNN	0.8091	0.8254	0.8038	0.8045	0.8038	0.8529	0.7958	0.7936
	SVM	0.7676	0.7700	0.7649	0.7655	0.7649	0.8760	0.8427	0.8424
	XGBoost	0.7925	0.7966	0.7894	0.7903	0.7894	0.8986	0.8261	0.8261
	LGBM	0.7635	0.7673	0.7601	0.7607	0.7601	0.8843	0.8177	0.8177
	CatBoost	0.8133	0.8312	0.8078	0.8085	0.8078	0.8933	0.8343	0.8345
	RF	0.7718	0.7889	0.7658	0.7653	0.7658	0.8797	0.7936	0.7923
Vit-base-patch32-224 Only Pathology	KNN	0.8216	0.8216	0.8206	0.8210	0.8206	0.8745	0.7793	0.7771
	SVM	0.7842	0.7870	0.7815	0.7822	0.7815	0.8771	0.8260	0.8259
	XGBoost	0.7718	0.7715	0.7707	0.7710	0.7707	0.8876	0.7931	0.7925
	LGBM	0.8216	0.8213	0.8210	0.8211	0.8210	0.9074	0.8177	0.8177
	CatBoost	0.8133	0.8173	0.8104	0.8114	0.8104	0.9043	0.7856	0.7839
	RF	0.7842	0.7908	0.7804	0.7811	0.7804	0.8915	0.7765	0.7758
Vit-base-patch16-224 Only MRI	KNN	0.8257	0.8394	0.8303	0.8250	0.8303	0.8751	0.9173	0.9172
	SVM	0.8714	0.8755	0.8739	0.8713	0.8739	0.9432	0.8213	0.8185
	XGBoost	0.8714	0.8742	0.8736	0.8714	0.8736	0.9495	0.7930	0.7930
	LGBM	0.8631	0.8659	0.8653	0.8631	0.8653	0.9447	0.8261	0.8261
	CatBoost	0.8714	0.8787	0.8747	0.8712	0.8747	0.9447	0.7768	0.7766
	RF	0.8838	0.8845	0.8851	0.8838	0.8851	0.9280	0.8596	0.8589
Vit-base-patch32-224 Only MRI	KNN	0.7967	0.8085	0.8010	0.7960	0.8010	0.8537	0.8682	0.8671
	SVM	0.8714	0.8732	0.8732	0.8714	0.8732	0.9298	0.7186	0.7185
	XGBoost	0.8382	0.8381	0.8388	0.8381	0.8388	0.9097	0.8596	0.8589
	LGBM	0.8631	0.8627	0.8630	0.8628	0.8630	0.9207	0.8843	0.8838
	CatBoost	0.8548	0.8551	0.8558	0.8547	0.8558	0.9089	0.8349	0.8339
	RF	0.8548	0.8546	0.8543	0.8544	0.8543	0.9175	0.8179	0.8174

**Table 5:** Classifier Performance in Breast Cancer Diagnosis with ViT-Based Multi-Image Fusion.

ViT Model	Classifier	Performance Evaluation Criteria							
		Accuracy	Precision	Recall	F-Score	AUC	AP	MCC	(κ)
Vit-base-patch16-224 Fusion	KNN	0.8340	0.8378	0.8314	0.8325	0.8314	0.8853	**0.9586**	**0.9586**
	SVM	0.8838	0.8851	0.8824	0.8832	0.8824	0.9582	0.9420	0.9420
	XGBoost	0.8589	0.8600	0.8575	0.8582	0.8575	0.9402	0.9178	0.9173
	LGBM	0.8755	0.8768	0.8741	0.8749	0.8741	0.9505	0.9337	0.9337
	CatBoost	**0.9046**	**0.9045**	**0.9053**	**0.9045**	**0.9053**	**0.9552**	0.9006	0.9006
	RF	0.8797	0.8795	0.8792	0.8794	0.8792	0.9555	0.9172	0.9171
Vit-base-patch32-224 Fusion	KNN	0.8423	0.8419	0.8424	0.8421	0.8424	0.9193	0.9509	0.9504
	SVM	**0.9253**	**0.9250**	**0.9255**	**0.9252**	**0.9255**	**0.9708**	0.9172	0.9171
	XGBoost	0.9087	0.9085	0.9093	0.9086	0.9093	0.9683	**0.9589**	**0.9586**
	LGBM	0.9170	0.9178	0.9161	0.9167	0.9161	0.9696	0.9424	0.9421
	CatBoost	0.8963	0.8960	0.8966	0.8962	0.8966	0.9622	0.9339	0.9338
	RF	0.8755	0.8761	0.8745	0.8750	0.8745	0.9553	0.9421	0.9420

**Note:** Bold values represent the highest value achieved for each evaluation metric.

## Data Availability

The original data presented in the study are openly available in https://github.com/mpbaydogan/breast-cancer-dataset-for-vit-models (accessed on 7 July 2026).
